# Design, Synthesis, Molecular Docking, and Antibacterial Evaluation of Some Novel Flouroquinolone Derivatives as Potent Antibacterial Agent

**DOI:** 10.1155/2014/897187

**Published:** 2014-12-09

**Authors:** Mehul M. Patel, Laxman J. Patel

**Affiliations:** ^1^Ramanbhai Patel College of Pharmacy, Charotar University of Science and Technology, CHARUSAT Campus, Changa, Petlad Taluka, Anand District, Gujarat 388 421, India; ^2^S. K. Patel College of Pharmaceutical Education and Research, Ganpat University, Kherva, Gujarat 382711, India

## Abstract

*Objective*. Quinolone moiety is an important class of nitrogen containing heterocycles widely used as key building blocks for medicinal agents. It exhibits a wide spectrum of pharmacophores and has bactericidal, antiviral, antimalarial, and anticancer activities. In view of the reported antimicrobial activity of various fluoroquinolones, the importance of the C-7 substituents is that they exhibit potent antimicrobial activities. Our objective was to synthesize newer quinolone analogues with increasing bulk at C-7 position of the main 6-fluoroquinolone scaffold to produce the target compounds which have potent antimicrobial activity. *Methods*. A novel series of 1-ethyl-6-fluoro-4-oxo-7-{4-[2-(4-substituted phenyl)-2-(substituted)-ethyl]-1-piperazinyl}-1,4-dihydroquinoline-3-carboxylic acid derivatives were synthesized. To understand the interaction of binding sites with bacterial protein receptor, the docking study was performed using topoisomerase II DNA gyrase enzymes (PDB ID: 2XCT) by Schrodinger's Maestro program. *In vitro* antibacterial activity of the synthesized compounds was studied and the MIC value was calculated by the broth dilution method. *Results*. Among all the synthesized compounds, some compounds showed potent antimicrobial activity. The compound **8g** exhibited good antibacterial activity. *Conclusion*. This investigation identified the potent antibacterial agents against certain infections.

## 1. Introduction

In recent years microbial infections are associated with high rates of attributable morbidity and mortality. Infections caused by microbial species are common in immune compromised patients and have significant treatment costs and mortality. The increasing rate of bacterial resistance to clinical antimicrobial agents and its impact on treatment of infectious diseases have begun to present a unique problem throughout the world. Drug resistant, multiple drug resistant (MDR), and extensively drug resistant (XDR) infectious bacterial pathogens put a greater risk on the population at large due to the risk of pandemic illness. Increasing complication is the fact that many antibacterial agents can induce mutations and resistance, often by different mechanisms. Methicillin-resistant* Staphylococcus aureus* (MRSA) and vancomycin-resistant* Enterococcus* (VRE) are the most important infections caused by bacteria which have been found worldwide in hospitals [1, 2]. The emerging resistance of some microbial species to some synthetic antimicrobial agents makes it necessary to continue the search for new antimicrobial agents. Quinolone moiety is an important class of nitrogen containing heterocycles widely used as key building blocks for medicinal agents. It exhibits a wide spectrum of pharmacophores and has bactericidal [3], antiviral [4], antimalarial [5], and anticancer [6] activities. Among these, flouroquinolone derivatives play an important role in the field of medicine. Fluoroquinolones are the most widely used antibacterial agents in modern therapy due to their broad spectrum and excellent oral bioavailability. In this work our interest is to study the antibacterial activity of novel 1-ethyl-6-fluoro-4-oxo-7-{4-[2-(4-substituted-phenyl)-2-(substituted)-ethyl]-1-piperazinyl}-1,4-dihydroquinoline-3-carboxylic acid derivatives. The present investigation deals with the clinically isolated different Gram-positive and Gram-negative bacteria against synthesized compounds and most of the tested compounds act as potent antibacterial agents. To understand the interactions of tested compounds at active sites of protein receptors, topoisomerase II DNA gyrase enzymes (PDB ID: 2XCT) molecular docking studies were also performed and reported in this paper.

## 2. Materials and Method

Melting points (°C, uncorrected) of all the synthesized compounds were checked in capillary tubes by using a digital melting point apparatus (Veego melting point apparatus). All the reactions were monitored by thin layer chromatography (TLC silica gel 0.25 mm, 60 G F254; eluting solvents were chloroform : methanol : hexane 9 : 0.5 : 0.5). All the compounds were characterized by FT-IR spectrometer (IR Prestige-21, Shimadzu, Japan) using KBr pellets; ^1^HNMR spectra were obtained in CDCl_3_ on Bruker Avance-II 400 MHz instrument and chemical shifts were measured as parts per million downfield from tetramethylsilane (TMS) as internal standard. Mass spectra were recorded on LCMS 2010 EV S Shimadzu mass spectrometer.

### 2.1. Procedure for Synthesis of 3-Chloro-4-fluoro Ethyl Anilinomethylene Malonate [7] (**2**)

A mixture of 3-chloro-4-fluoro aniline (**1**) (1.4 g, 10 mmol) and diethyl ethoxymethylene malonate (2.16 g, 10 mmol) was taken in glass microwave vessel and kept in microwave at 80% intensity for 60 sec (3 times). Ethanol was removed by vacuum pump. The crude solid was dried and recrystallized from n-hexane (yield 90%; melting point 55-56°C (55–57°C); *R*_*f*_ value 0.73 (mobile phase: hexane : ethyl acetate 2 : 1)).

### 2.2. Procedure for Synthesis of 7-Chloro-6-fluoro-1,4-dihydro-4-oxoquinoline-3-carboxylic Acid Ethyl Ester [8] (**3**)

The 3-chloro-4-fluoro ethyl anilinomethylene malonate (**2**) (1 g, 3.17 mmol) was added to diphenyl ether (10 mL) and refluxed for 2 to 3 hrs. The mixture was cooled, filtered, and dried. The crude solid obtained was purified by recrystallization from hexane (yield 85%; melting point 288–290°C (290°C); *R*_*f*_ value 0.68 (mobile phase: chloroform : methanol 9 : 1)).

### 2.3. Procedure for Synthesis of 7-Chloro-1-ethyl-6-fluoro-1,4-dihydro-4-oxoquinoline-3-carboxylic Acid Ethyl Ester [8] (**4**)

The mixture of 7-chloro-6-fluoro-1,4-dihydro-4-oxoquinoline-3-carboxylic acid ethyl ester (**3**) (0.50 g, 1.86 mmol), ethyl iodide (1.45 g, 9.3 mmol), potassium carbonate (6.42 mg, 46.5 mmol), and dimethyl formamide (20 mL) was taken in 50 mL round bottom flask and heated at 110–120°C for 5 to 6 hrs. Ice cold water was added to the reaction mixture. The crude solid obtained was purified and recrystallized from ethanol (yield 75%; melting point 139–141°C (142°C); *R*_*f*_ value 0.93 (mobile phase: chloroform : methanol 9 : 1)).

### 2.4. Procedure for Synthesis of 7-Chloro-1-ethyl-6-fluoro-1,4-dihydro-4-oxoquinoline-3-carboxylic Acid [8] (**5**)

7-Chloro-1-ethyl-6-fluoro-1,4-dihydro-4-oxoquinoline-3-carboxylic acid ethyl ester (**4**) (200 mg, 0.674 mmol) and sodium hydroxide (2 N, 2 mL) were taken into 50 mL round bottom flask and refluxed for 2 to 3 hrs. The resulting mixture was filtered, filtrate was neutralized by acetic acid (pH 2), and separated solid was collected and washed with water. The solid was recrystallized with methanol (yield 62%; melting point 280–282°C (284°C); *R*_*f*_ value 0.47 (mobile phase: chloroform : methanol 9 : 1)).

### 2.5. Procedure for Synthesis of 1-Ethyl-6-fluoro-1,4-dihydro-4-oxo-7-(1-piperazinyl)-quinoline-3-carboxylic Acid [8] (**6**)

In 50 mL glass microwave vessel, a mixture of** 5** (215.6 mg, 0.80 mmol), piperazine (75.8 mg, 0.88 mmol), triethylamine (165.6 mg, 2.40 mmol), and dimethyl formamide (1 mL) was irradiated in microwave at 80% intensity for a given interval of time (60 + 60 + 30 sec). The mixture was evaporated to dryness. Water was added to the reaction mixture and the residue was filtered off, washed with water, dried, and recrystallized from dichloromethane and methanol to give** 6** (yield 62%; melting point 223–225°C (226°C); *R*_*f*_ value 0.38 (mobile phase: chloroform : methanol 2 : 3)).

### 2.6. General Procedure for Synthesis of 1-Ethyl-6-fluoro-4-oxo-7-{4-[2-(4-substituted phenyl)-2-(substituted)-ethyl]-1-piperazinyl}-1,4-dihydroquinoline-3-carboxylic Acids (**8**)

**Scheme 1:**
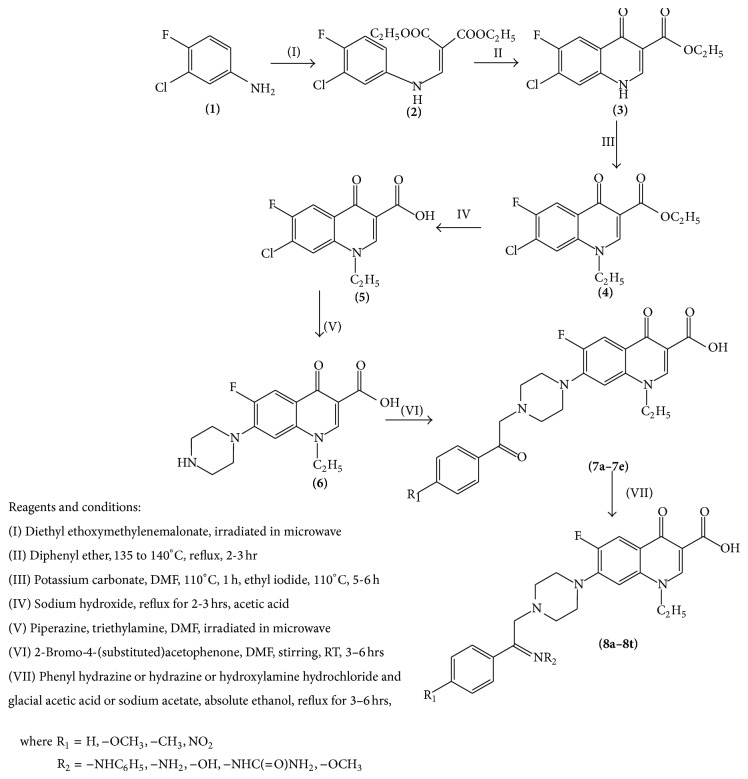
Synthesis of 1-ethyl-6-fluoro-4-oxo-7-{4-[2-(4-substituted phenyl)-2-(substituted)-ethyl]-1-piperazinyl}-1,4-dihydroquinoline-3-carboxylic acid.

A mixture of 1-ethyl-6-fluoro-4-oxo-7-(piperazin-1-yl)-1,4-dihydroquinoline-3-carboxylic acid (1 mmol) (**6**), sodium bicarbonate (2 mmol), and 2-bromo-4-(Substituted)acetophenone (1 mmol) in dimethyl formamide (40 mL) was stirred at room temperature for 3 to 6 hrs. The reaction mixture was poured into ice water (50 mL) [9]. The separated solid of** 7** was collected by filteration, dried, and purified by recrystallization from methanol-chloroform. Reaction of** 7** with phenylhydrazine, hydrazine, hydroxylamine, semicarbazide, and methoxylamine hydrochloride, respectively, in ethanol with glacial acetic acid for 3 to 6 hrs at 110–120°C gave the corresponding compounds (**8a**–**8t**) (shown in Scheme 1) in 45–74% overall yield (shown in Table 1).

### 2.7. Spectral Data

#### 2.7.1. 1-Ethyl-6-fluoro-4-oxo-7-{4-[2-phenyl-2-(phenyl-hydrazono)-ethyl]-piperazin-1-yl}-1,4-dihydro-quinoline-3-carboxylic Acid (**8a**)

IR (KBr): 1629, 1718 (C=O), 3049 (C–H) cm^−1^; ^1^H NMR (500 MHz, CDCl_3_) *δ* 1.58 (t, 3H, –CH_3_), *δ* 4.31 (q, 2H, –CH_2_), *δ* 2.84 & *δ* 3.38 (m, 8H, Piperazinyl), *δ* 3.87 (s, 2H, N–CH_2_), *δ* 6.83–8.10 (m, 12H, Aromatic $\underline{\text{H}}$), *δ* 8.69 (s, 1H, C=C–H), *δ* 10.83 (s, 1H, N–NH–Ph), *δ* 15.08 (s, 1H, COOH); MASS (EI)* m/z*: Calcd for C_30_H_30_F_1_N_5_O_3_: 527 Found: 528.

#### 2.7.2. 1-Ethyl-6-fluoro-4-oxo-7-[4-(2-phenyl-2-hydrazono-ethyl)-piperazin-1-yl]-4-1,4-dihydro-quinoline-3-carboxylic Acid (**8b**)

IR (KBr): 1624, 1701 (C=O), 3369 (N–H) cm^−1^; ^1^H NMR (500 MHz, CDCl_3_) *δ* 1.25 (t, 3H, –CH_3_), *δ* 4.32 (q, 2H, –CH_2_), *δ* 2.80 & *δ* 3.34 (m, 8H, Piperazin), *δ* 3.75 (s, 2H, N–CH_2_), *δ* 6.87–8.09 (m, 7H, Aromatic $\underline{\text{H}}$), *δ* 8.68 (s, 1H, C=C–H), *δ* 15.09 (s, 1H, COOH); MASS (EI)* m/z*: Calcd for C_24_H_26_F_1_N_5_O_3_: 451 Found: 452.

#### 2.7.3. 1-Ethyl-6-fluoro-4-oxo-7-[4-(2-phenyl-2-hydroxy-iminoethyl)-piperazin-1-yl]-1,4-dihydro-quinoline-3-carboxylic Acid (**8c**)

IR (KBr): 1629, 1896 (C=O), 3221 (O–H); ^1^H NMR (500 MHz, CDCl_3_) *δ* 1.58 (t, 3H, –CH_3_), *δ* 4.31 (q, 2H, –CH_2_), *δ* 2.77 & *δ* 3.36 (m, 8H, Piperazinyl), *δ* 3.88 (s, 2H, N–CH_2_), *δ* 6.80–8.10 (m, 7H, Aromatic $\underline{\text{H}}$), *δ* 8.69 (s, 1H, C=C–H), *δ* 15.08 (s, 1H, COOH); MASS (EI)* m/z*: Calcd for C_24_H_25_F_1_N_4_O_4_: 452 Found: 453.

#### 2.7.4. 1-Ethyl-6-fluoro-4-oxo-7-[4-(2-phenyl-2-carbamoylhydrazinylidene-ethyl)-piperazin-1-yl]-1,4-dihydro-quinoline-3-carboxylic Acid (**8d**)

IR (KBr): 1685 (C=O), 3037 (C–H) cm^−1^; ^1^H NMR (500 MHz, CDCl_3_) *δ* 4.25 (q, 2H, –CH_2_) *δ* 2.72 & *δ* 3.32 (m, 8H, Piperazinyl), *δ* 3.74 (s, 2H, N–CH_2_), *δ* 6.79–8.04 (m, 7H, Aromatic $\underline{\text{H}}$), *δ* 8.63 (s, 1H, C=C–H), *δ* 10.95 (s, 1H, –N–NH–CO–), *δ* 14.97 (s, 1H, COOH); MASS (EI)* m/z*: Calcd for C_25_H_27_F_1_N_6_O_4_: 494 Found: 495.

#### 2.7.5. 1-Ethyl-6-fluoro-4-oxo-7-[4-(2-phenyl-2-methoxy-iminoethyl)-piperazin-1-yl]-1,4-dihydro-quinoline-3-carboxylic Acid (**8e**)

IR (KBr): 1633, 1718 (C=O), 2937 (C–H) cm^−1^; ^1^H NMR (500 MHz, CDCl_3_) *δ* 4.25 (q, 2H, –CH_2_) *δ* 2.72 & *δ* 3.28 (m, 8H, Piperazinyl), *δ* 3.70 (s, 2H, N–CH_2_), *δ* 6.77–8.27 (m, 7H, Aromatic $\underline{\text{H}}$), *δ* 8.61 (s, 1H, C=C–H), *δ* 14.97 (s, 1H, COOH); MASS (EI)* m/z*: Calcd for C_25_H_27_FN_4_O_4_: 466 Found: 467.

#### 2.7.6. 1-Ethyl-6-fluoro-4-oxo-7-{4-[2-(4-methoxyphenyl)-2-(phenyl-hydrazono)-ethyl]-piperazin-1-yl}-1,4-dihydro-quinoline-3-carboxylic Acid (**8f**)

IR (KBr): 1625, 1728 (C=O), 3057 (C–H) cm^−1^; ^1^H NMR (500 MHz, CDCl_3_) *δ* 1.58 (t, 3H, –CH_3_), *δ* 4.32 (q, 2H, –CH_2_), *δ* 2.83 & *δ* 3.37 (m, 8H, Piperazinyl), *δ* 3.83 (s, 2H, N–CH_2_), *δ* 3.84 (s, 3H, –OCH_3_), *δ* 6.82–8.10 (m, 11H, Aromatic $\underline{\text{H}}$), *δ* 8.68 (s, 1H, C=C–H), *δ* 10.69 (s, 1H, N–NH–Ph) *δ* 15.07 (s, 1H, COOH); MASS (EI)* m/z*: Calcd for C_31_H_32_F_1_N_5_O_4_: 557 Found: 558.

#### 2.7.7. 1-Ethyl-6-fluoro-4-oxo-7-[4-(2-(4-methoxyphenyl)-2-hydrazono-ethyl)-piperazin-1-yl]-4-1,4-dihydro-quinoline-3-carboxylic Acid (**8g**)

IR (KBr): 1624, 1718 (C=O), 3408 (N–H) cm^−1^; ^1^H NMR (500 MHz, CDCl_3_) *δ* 4.31 (q, 2H, –CH_2_) *δ* 2.85 & *δ* 3.34 (m, 8H, Piperazinyl), *δ* 3.72 (s, 2H, N–CH_2_), *δ* 3.83 (s, 3H, –OCH_3_), *δ* 6.74–8.09 (m, 6H, Aromatic $\underline{\text{H}}$), *δ* 8.68 (s, 1H, C=C–H), *δ* 15.09 (s, 1H, COOH); MASS (EI)* m/z*: Calcd for C_25_H_28_F_1_N_5_O_4_: 481 Found: 482.

#### 2.7.8. 1-Ethyl-6-fluoro-4-oxo-7-[4-(2-(4-methoxyphenyl)-2-hydroxy-iminoethyl)-piperazin-1-yl]-1,4-dihydro-quinoline-3-carboxylic Acid (**8h**)

IR (KBr): 1695, 1716 (C=O), 3271 (OH) cm^−1^; ^1^H NMR (500 MHz, CDCl_3_) *δ* 4.31 (q, 2H, –CH_2_) *δ* 2.75 & *δ* 3.35 (m, 8H, Piperazinyl), *δ* 3.88 (s, 2H, N–CH_2_), *δ* 3.84 (s, 3H, –OCH_3_), *δ* 6.90–8.11 (m, 6H, Aromatic $\underline{\text{H}}$), *δ* 8.69 (s, 1H, C=C–H), *δ* 15.08 (s, 1H, COOH); MASS (EI)* m/z*: Calcd for C_25_H_27_F_1_N_4_O_5_: 482 Found: 483.

#### 2.7.9. 1-Ethyl-6-fluoro-4-oxo-7-[4-(2-(4-methoxyphenyl)-2-carbamoylhydrazinylidene-ethyl)-piperazin-1-yl]-1,4-dihydro-quinoline-3-carboxylic Acid (**8i**)

IR (KBr): 1674, 1720 (C=O), 3016, 3485 (N–H) cm^−1^; ^1^H NMR (500 MHz, CDCl_3_) *δ* 4.33 (q, 2H, –CH_2_), *δ* 2.83 & *δ* 3.31 (m, 8H, Piperazinyl), *δ* 3.71 (s, 2H, N–CH_2_), *δ* 3.83 (s, 3H, –OCH_3_), *δ* 6.77–8.09 (m, 6H, Aromatic $\underline{\text{H}}$), *δ* 8.67 (s, 1H, C=C–H), *δ* 15.05 (s, 1H, COOH); MASS (EI)* m/z*: Calcd for C_26_H_29_F_1_N_6_O_5_: 524 Found: 525.

#### 2.7.10. 1-Ethyl-6-fluoro-4-oxo-7-[4-(2-(4-methoxyphenyl)-2-methoxy-iminoethyl)-piperazin-1-yl]-1,4-dihydro-quinoline-3-carboxylic Acid (**8j**)

IR (KBr): 1625, 1718 (C=O), 2939 (C–H) cm^−1^; ^1^H NMR (500 MHz, CDCl_3_) *δ* 4.21 (q, 2H, –CH_2_) *δ* 2.66 & *δ* 3.21 (m, 8H, Piperazinyl), *δ* 3.71 (s, 2H, N–CH_2_), *δ* 3.94 (s, 3H, –OCH_3_ Side Chain) *δ* 3.85 (s, 3H, –OCH_3_), *δ* 6.73–8.01 (m, 7H, Aromatic $\underline{\text{H}}$), *δ* 8.60 (s, 1H, C=C–H), *δ* 14.93 (s, 1H, COOH); MASS (EI)* m/z*: Calcd for C_26_H_29_FN_4_O_5_: 496 Found: 497.

#### 2.7.11. 1-Ethyl-6-fluoro-4-oxo-7-{4-[2-(4-methylphenyl)-2-(phenyl-hydrazono)-ethyl]-piperazin-1-yl}-1,4-dihydro-quinoline-3-carboxylic Acid (**8k**)

IR (KBr): 1629, 1730 (C=O), 3062 (C–H) cm^−1^; ^1^H NMR (500 MHz, CDCl_3_) *δ* 1.58 (t, 3H, –CH_3_), *δ* 4.32 (q, 2H, –CH_2_), *δ* 2.83 & *δ* 3.37 (m, 8H, Piperazinyl), *δ* 3.84 (s, 2H, N–CH_2_), *δ* 2.38 (s, 3H, –CH_3_), *δ* 6.82–8.11 (m, 11H, Aromatic $\underline{\text{H}}$), *δ* 8.68 (s, 1H, C=C–H), *δ* 10.76 (s, 1H, N–NH–Ph) *δ* 15.07 (s, 1H, COOH); MASS (EI)* m/z*: Calcd for C_31_H_32_F_1_N_5_O_3_: 541 Found: 542.

#### 2.7.12. 1-Ethyl-6-fluoro-4-oxo-7-[4-(2-(4-methylphenyl)-2-hydrazono-ethyl)-piperazin-1-yl]-4-1,4-dihydro-quinoline-3-carboxylic Acid (**8l**)

IR (KBr): 1618, 1701 (C=O), 3387 (N–H) cm^−1^; ^1^H NMR (500 MHz, CDCl_3_) *δ* 4.32 (q, 2H, –CH_2_) *δ* 2.78 & *δ* 3.33 (m, 8H, Piperazinyl), *δ* 3.72 (s, 2H, N–CH_2_), *δ* 2.39 (s, 3H, –CH_3_), *δ* 6.80–8.09 (m, 6H, Aromatic $\underline{\text{H}}$), *δ* 8.68 (s, 1H, C=C–H), *δ* 15.07 (s, 1H, COOH); MASS (EI)* m/z*: Calcd for C_25_H_28_F_1_N_5_O_3_: 465 Found: 466.

#### 2.7.13. 1-Ethyl-6-fluoro-4-oxo-7-[4-(2-(4-methylphenyl)-2-hydroxy-iminoethyl)-piperazin-1-yl]-1,4-dihydro-quinoline-3-carboxylic Acid (**8m**)

IR (KBr): 1629, 1718 (C=O), 3277 (O–H) cm^−1^; ^1^H NMR (500 MHz, CDCl_3_) *δ* 4.31 (q, 2H, –CH_2_) *δ* 2.77 & *δ* 3.36 (m, 8H, Piperazinyl), *δ* 3.86 (s, 2H, N–CH_2_), *δ* 2.38 (s, 3H, –CH_3_), *δ* 6.83–8.12 (m, 6H, Aromatic $\underline{\text{H}}$), *δ* 8.69 (s, 1H, C=C–H), *δ* 15.05 (s, 1H, COOH); MASS (EI)* m/z*: Calcd for C_25_H_27_F_1_N_4_O_4_: 466 Found: 467.

#### 2.7.14. 1-Ethyl-6-fluoro-4-oxo-7-[4-(2-(4-methylphenyl)-2-carbamoylhydrazinylidene-ethyl)-piperazin-1-yl]-1,4-dihydro-quinoline-3-carboxylic Acid (**8n**)

IR (KBr): 1701, 1718 (C=O), 3032 (C–H) cm^−1^; ^1^H NMR (500 MHz, CDCl_3_) *δ* 4.33 (q, 2H, –CH_2_) *δ* 2.78 & *δ* 3.35 (m, 8H, Piperazinyl), *δ* 3.13 (s, 2H, N–CH_2_), *δ* 2.39 (s, 3H, –CH_3_), *δ* 6.80–8.03 (m, 6H, Aromatic $\underline{\text{H}}$), *δ* 8.68 (s, 1H, C=C–H), *δ* 10.99 (s, 1H, –N–NH–CO–), *δ* 15.09 (s, 1H, COOH); MASS (EI)* m/z*: Calcd for C_26_H_29_F_1_N_6_O_4_: 508 Found: 509.

#### 2.7.15. 1-Ethyl-6-fluoro-4-oxo-7-[4-(2-(4-methylphenyl)-2-methoxy-iminoethyl)-piperazin-1-yl]-1,4-dihydro-quinoline-3-carboxylic Acid (**8o**)

IR (KBr): 1626, 1732 (C=O), 2937 (C–H) cm^−1^; ^1^H NMR (500 MHz, CDCl_3_) *δ* 1.49 (t, 3H, –CH_3_) *δ* 4.22 (q, 2H, –CH_2_), *δ* 2.65 & *δ* 3.21 (m, 8H, Piperazinyl), *δ* 3.38 (s, 2H, N–CH_2_), *δ* 3.91 (s, 3H, –OCH_3_ Side Chain), *δ* 2.29 (s, 3H, –CH_3_), *δ* 6.70–7.97 (m, 6H, Aromatic $\underline{\text{H}}$), *δ* 8.58 (s, 1H, C=C–H), *δ* 15.06 (s, 1H, COOH); MASS (EI)* m/z*: Calcd for C_26_H_29_F_1_N_4_O_4_: 480 Found: 481.

#### 2.7.16. 1-Ethyl-6-fluoro-4-oxo-7-{4-[2-(4-nitrophenyl)-2-(phenyl-hydrazono)-ethyl]-piperazin-1-yl}-1,4-dihydro-quinoline-3-carboxylic Acid (**8p**)

IR (KBr): 1629, 1707 (C=O), 3336 (N–H) cm^−1^; ^1^H NMR (500 MHz, CDCl_3_) *δ* 4.23 (q, 2H, –CH_2_) *δ* 2.77 & *δ* 3.32 (m, 8H, Piperazinyl), *δ* 3.83 (s, 2H, N–CH_2_), *δ* 2.38 (s, 3H, –CH_3_), *δ* 6.76–8.17 (m, 10H, Aromatic $\underline{\text{H}}$), *δ* 8.62 (s, 1H, C=C–H), *δ* 11.08 (s, 1H, N–NH–Ph), *δ* 14.93 (s, 1H, COOH); MASS (EI)* m/z*: Calcd for C_30_H_29_FN_6_O_5_: 572 Found: 573.

#### 2.7.17. 1-Ethyl-6-fluoro-4-oxo-7-[4-(2-(4-nitrophenyl)-2-hydrazono-ethyl)-piperazin-1-yl]-4-1,4-dihydro-quinoline-3-carboxylic Acid (**8q**)

IR (KBr): 1691, 1726 (C=O), 3394 (N–H) cm^−1^; ^1^H NMR (500 MHz, CDCl_3_) *δ* 4.22 (q, 2H, –CH_2_) *δ* 2.67 & *δ* 3.21 (m, 8H, Piperazinyl), *δ* 3.83 (s, 2H, N–CH_2_), *δ* 6.72–7.98 (m, 6H, Aromatic $\underline{\text{H}}$), *δ* 8.58 (s, 1H, C=C–H), *δ* 15.06 (s, 1H, COOH); MASS (EI)* m/z*: Calcd for C_24_H_25_FN_6_O_5_: 496 Found: 497.

#### 2.7.18. 1-Ethyl-6-fluoro-4-oxo-7-[4-(2-(4-nitrophenyl)-2-hydroxy-iminoethyl)-piperazin-1-yl]-1,4-dihydro-quinoline-3-carboxylic Acid (**8r**)

IR (KBr): 1631, 1714 (C=O), 3199 (OH) cm^−1^; ^1^H NMR (500 MHz, CDCl_3_) *δ* 4.21 (q, 2H, –CH_2_) *δ* 2.69 & *δ* 3.23 (m, 8H, Piperazinyl), *δ* 3.81 (s, 2H, N–CH_2_), *δ* 6.87–8.08 (m, 6H, Aromatic $\underline{\text{H}}$), *δ* 8.58 (s, 1H, C=C–H), *δ* 15.06 (s, 1H, COOH); MASS (EI)* m/z*: Calcd for C_24_H_24_FN_5_O_6_: 497 Found: 498.

#### 2.7.19. 1-Ethyl-6-fluoro-4-oxo-7-[4-(2-(4-nitrophenyl)-2-carbamoylhydrazinylidene-ethyl)-piperazin-1-yl]-1,4-dihydro-quinoline-3-carboxylic Acid (**8s**)

IR (KBr): 1691, 1726 (C=O), 3062 (C–H) cm^−1^; ^1^H NMR (500 MHz, CDCl_3_) *δ* 4.21 (q, 2H, –CH_2_), *δ* 2.67 & *δ* 3.26 (m, 8H, Piperazinyl), *δ* 3.84 (s, 2H, N–CH_2_), *δ* 6.75–8.01 (m, 6H, Aromatic $\underline{\text{H}}$), *δ* 8.57 (s, 1H, C=C–H), *δ* 10.97 (s, 1H, –N–NH–CO–), *δ* 15.01 (s, 1H, COOH); MASS (EI)* m/z*: Calcd for C_25_H_26_FN_7_O_6_: 539 Found: 540.

#### 2.7.20. 1-Ethyl-6-fluoro-4-oxo-7-[4-(2-(4-nitrophenyl)-2-methoxy-iminoethyl)-piperazin-1-yl]-1,4-dihydro-quinoline-3-carboxylic Acid (**8t**)

IR (KBr): 1629, 1722 (C=O), 2939 (C–H) cm^−1^; ^1^H NMR (500 MHz, CDCl_3_) *δ* 1.50 (t, 3H, –CH_3_) *δ* 4.23 (q, 2H, –CH_2_), *δ* 2.67 & *δ* 3.21 (m, 8H, Piperazinyl), *δ* 3.39 (s, 2H, N–CH_2_), *δ* 3.85 (s, 3H, –OCH_3_ Side Chain), *δ* 6.72–8.20 (m, 6H, Aromatic $\underline{\text{H}}$), *δ* 8.59 (s, 1H, C=C–H), *δ* 15.03 (s, 1H, COOH); MASS (EI)* m/z*: Calcd for C_25_H_26_FN_5_O_6_: 511 Found: 512.

### 2.8. Molecular Docking Studies

Molecular docking is an important computational technique in structural biology and computer aided drug design. The major objective of molecular docking is to evaluate the feasible binding geometries of a putative ligand with a target protein of known three-dimensional structure.

To understand the interaction of all the synthesized molecules (**8a–8t**) with topoisomerase II DNA gyrase enzymes, the crystal structure of topoisomerase II was downloaded from the Protein Data Bank (PDB ID: 2XCT) and molecular docking studies were performed using the Glide program [10, 11] (version 6.3, Schrodinger, LLC, New York, 2014). To analyze the docking results and execute the protocol, the Maestro user interface (version 9.7, Schrodinger, LLC, New York, 2014) was employed. Docking was performed using the XP (Extra Precision Mode) docking protocol.

#### 2.8.1. Preparation of Protein

The structure of topoisomerase II DNA gyrase enzyme (PDB ID: 2XCT) was downloaded from the Protein Data Bank and imported and prepared by a multistep process through the protein preparation wizard of Maestro (version 9.7). The protocol was especially used to obtain the optimised and minimised energy conformation of the protein. Firstly, the bond order in the protein was assigned; hydrogen atoms were added and the water molecules which did not participate in interactions were removed. Following the above steps of preparation, the protein was subjected to energy minimisation using Schrodinger implementation of OPLS-2005 force field with implicit solvation.

#### 2.8.2. Preparation of Ligands

The ligands were prepared using the LigPrep 3.0 [12] module of the Schrodinger suite [13] using Merck molecular force field (MMFF). MMFF was used for 2D to 3D conversion of ligand molecules while the optimized potential for liquid simulation (OPLS) force field was used for conformation analysis through ConfGen [14].

### 2.9. Active Site Prediction

Active site is a pocket pouch present on the protein structure that has the tendency to accept the ligand molecules within it. The Sitemap applies theoretical methods and predicts the most accurate binding site. The OPLS-AA force field generates site points, possible for ligand interaction within the protein. Sitemap gives an idea about positions favorable for a donor, acceptor, and hydrophobic group to be present in the receptor. The maps were very useful for analysing the interactions of ligands with the receptor. In order to find the active site volume, surface area, and character of binding sites of the enzymes (2XCT), Sitemap was performed by choosing the top ranked potential receptor binding sites and the other parameters were kept as default. OPLS-2005 was used as the force field. Five sites with different site scores were obtained as output and the site with the highest site score was selected.

Docking studies on LigPrep treated ligands were carried out to predict the binding pocket of 2XCT using the docking program, Glide. Glide used a series of hierarchical filters to search for possible locations for the ligand in the active site region of the receptor. For the grid-based ligand docking, the receptor grid generation file was used. For protein structure, a grid box of 30 × 30 × 30 Å with a default inner box (10 × 10 × 10 Å) was centered on the corresponding ligand. The receptor grid was defined as an enclosing box at the centroid of the cocrystallized ligand (i.e., 2XCT) to include the cofactor and substrate binding sites. In the initial Glide docking stage, a softened potential docking with the van der Waals radii scaling of 0.7 for the proteins was performed to retain the maximum number of 20 poses per ligand. Residues within 5.0 Å of ligand poses were kept free to move in the Prime refinement step, and the side chains were further minimized. Then single low energy 3D structure of ligands with correct chiralities was docked with the binding site using the “Extra Precision” Glide algorithm in Schrodinger.

### 2.10. *In Vitro* Antibacterial Activity

All the synthesized compounds were studied for their antibacterial activity against two clinically isolated Gram-positive strains (*S. aureus and S. pneumoniae*) and two Gram-negative strains (*P. aeruginosa and Escherichia coli*) using conventional broth dilution method. The minimum inhibitory concentration (MIC) values were calculated by comparison to norfloxacin as the reference bacterial drug and they are shown in Table 2.

All the cultures were prepared by Mueller-Hinton agar and the turbidity of all the bacterial cultures was adjusted to 0.5 McFarland standard by preparing bacterial suspension of 3–5 well-isolated colonies of the same morphological type selected from an agar plate culture. The cultures were further diluted 1000-fold to get an inoculum size of 1 × 10^6^ CFU. The synthesized compounds and standard bacterial drugs (50 mg) were dissolved in dimethyl sulphoxide (DMSO) (0.5 mL) and the solution was diluted with water (4.5 mL) to get a stock solution of 10,000 mg/L of each compound. Further progressive double dilution with melted Mueller-Hinton agar was performed to obtain the required concentrations of 100, 50, 25, 12.5, 6.25, and 1 *μ*g/mL [15, 16]. To ensure that the solvent had no effect on the bacterial growth, a control test was performed with a test medium supplemented with DMSO at the same dilution as that used in the experiment. Incubation of the cultures was done at 37°C for 24 hrs and comparison with blank was done in terms of turbidity which is due to microbial growth.

## 3. Results and Discussion

### 3.1. Chemistry

1-Ethyl-6-fluoro-4-oxo-7-{4-[2-(4-substituted phenyl)-2-oxoethyl]-1-piperazinyl}-1,4-dihydroquinoline-3-carboxylic acid (**7**) was prepared according to the literature method. Our synthetic route to target compounds (**8a–8t**) is presented in Scheme 1. The 1-ethyl-6-fluoro-4-oxo-7-{4-[2-(4-substituted phenyl)-2-oxoethyl]-1-piperazinyl}-1,4-dihydroquinoline-3-carboxylic acid derivatives (**7**) react with various amines to form target compounds. The structures were characterized by spectral techniques. In general, in FTIR spectra, characteristic N–H stretching peaks near 3269–3415 cm^−1^ and C=O stretching peaks near 1622–1732 cm^−1^ are shown. The ^1^H-NMR of synthesized derivatives showed multiple signals corresponding to resonance of fluoroquinolone protons, at two m, 8H in the region of *δ* 2.64 to 3.83 corresponding to piperazine hydrogen. The proton of the N–CH_2_ group exhibited a singlet in the region of *δ* 3.65 to 3.91. Aromatic protons were resonating as multiple in the region of *δ* 6.8 to 8.19. The proton of O–H of carboxylic acid exhibited a singlet in the region of *δ* 14.72 to 15.09. The proton of the C–H (alkenyl) of quinolone ring exhibited a singlet in the region of *δ* 8.58 to 8.80. All the compounds were characterized by mass spectral analysis. Compounds show that M^+^ peak corresponds to the molecular weight.

### 3.2. Biology

#### 3.2.1. *In Vitro* Antibacterial Studies of Fluoroquinolone Derivatives

All the synthesized compounds were tested against two Gram-positive and two Gram-negative bacteria. All the compounds (**8a–8t**) exhibited good bacterial activity against Gram-positive bacteria.

*Against Gram-Positive Strain*. Compounds** 8g**,** 8h**,** 8i**,** 8n**,** 8r**, and** 8s **have shown antibacterial activity with MIC value in the range of 6.25–25 *μ*g/mL against Gram-positive* S. aureus and S. pneumonia.* Compounds** 8l** and** 8q **have shown antibacterial activity with MIC value in the range of 12.5–25 *μ*g/mL against Gram-positive* S. aureus and S. pneumonia*. The Remaining compounds have shown antibacterial activity with MIC value in the range of 25 to ≥100 *μ*g/mL against Gram-positive* S. aureus and S. pneumonia.*

*Against Gram-Negative Strain*. Compounds** 8i**,** 8n**,** 8q**, and** 8s** have shown potent antibacterial activity with MIC value in the range of 6.25–50 *μ*g/mL against Gram-negative* E. coli *and* P. aeruginosa*. The remaining compounds have shown antibacterial activity with MIC value in the range of 12.5–50 *μ*g/mL against Gram-negative* E. coli *and ≥100 *μ*g/mL against Gram-negative* P. aeruginosa.* It was observed that** 8i**,** 8l**,** 8n**,** 8q**, and** 8s** have shown potent antibacterial activity against Gram-positive* S. aureus and S. pneumonia *and against Gram-negative* E. coli *strains and* P. aeruginosa*.

In terms of structure activity relationship, results suggest that compounds having 4-nitro and 4-methoxy on phenyl ring at C-7 piperazinyl side chain exhibited the most potent antibacterial activity. It was also observed that compounds with imino nitrogen substituted with a semicarbazide or amine (*R*_2_ = –NHCONH_2_ and NH_2_) exhibited potent antibacterial activity.

Compounds** 8g**,** 8h**,** 8i**,** 8n**,** 8r**,** 8s**,** 8l**, and** 8q** were found to be active as antibacterial agents. The pharmacological data indicated that flouroquinolone derivatives showed similar or potent activity against* S. aureus*,* S. pneumonia*,* E. coli*, and* P. aeruginosa* as compared with norfloxacin.

### 3.3. Molecular Docking Studies of Derivatives

To understand the interaction of all the synthesized molecules (**8a–8t**) with topoisomerase II DNA gyrase enzymes, the crystal structure of topoisomerase II was downloaded from Protein Data Bank (PDB ID: 2XCT) and the molecular docking studies were performed using the Glide program. The protein ligand interaction plays a significant role in structural based drug designing. In this approach, H-bonding, Glide energy score, Emodel, and *G*-score are kept as a support for the present work.

The preferred ligands** 8g**,** 8i**,** 8e**,** 8c**,** 8s**,** 8q**,** 8h**,** 8n**, and** 8a **had a *G*-score value of −8.51 to −7.43. The *G*-score values of the above mentioned standard compound (norfloxacin) were comparatively lesser than their analogues with the scoring of −7.29.

The minimum Glide energy required for the formation of complex between ligand and the receptor indicates excellent binding affinity. Very low energy indicates that the ligand is buried in the cavity of the receptor [17]. The Glide binding energy of the fluroquinolone analogues as ligands was found to be −54.38 to −74.83. Standard compounds such as norfloxacin possessed Glide energy −54.23.

In the present investigation, the nine fluoroquinolone analogues ligands had high quality binding affinity with the enzyme. Hydrogen bond interaction can act either as antagonist or as agonist for a ligand with receptor. On analyzing the docking results, all the seven compounds had H-bond interactions with the receptor indicating that the ligand binding affinity increased together with the number of the H-bond interactions. All nine ligands had a significant number of H-bond interactions with the enzyme.

Emodel had a more significant weighting of the force field components (electrostatic and van der Waals energies), which made it well suited for comparing conformers. Therefore, Glide uses Emodel to pick the “best” pose of a ligand (pose selection) and then ranks these best poses against one another with Glide score. A low Emodel value indicates a good binding affinity between protein and ligand. Glide Emodel values of −119.918 to −84.85, for the nine best fluroquinolone analogues, were calculated (Table 3) indicating that these compounds engage in energetically more favourable interactions than norfloxacin (Emodel score −83.0725, Table 3) within the active site.

**Figure 1:**
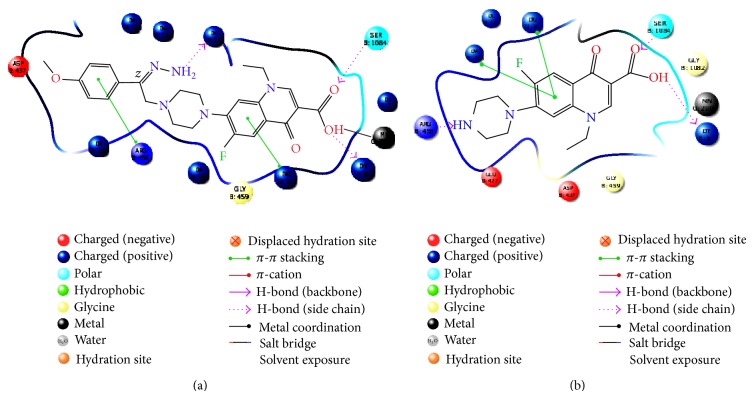
2D interaction diagram for the complex protein-ligand: (a) after docking interaction between 2XCT and** 8g** and (b) interaction between 2XCT and norfloxacin.

**Figure 2:**
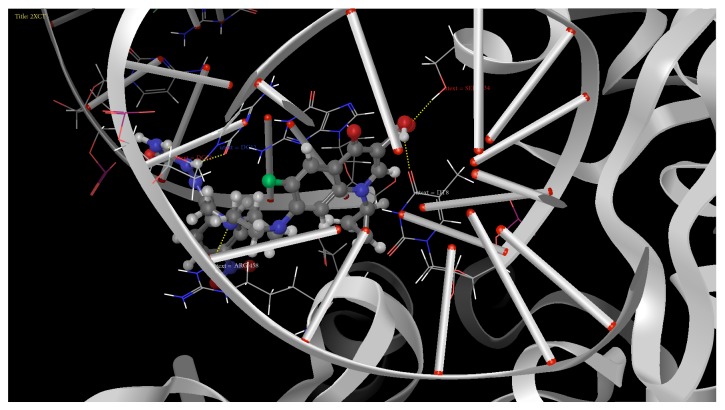
H-bond interactions between target protein 2XCT and studied compounds. Binding interaction of docked compound (**8g**) with topoisomerase II DNA gyrase enzymes.

From the docking study we predicted that fluoroquinolone analogues (**8g**,** 8i**,** 8e**,** 8c**,** 8s**,** 8q**,** 8h**,** 8n**, and** 8a**) possess better antibacterial activity than the standard drugs by having good binding affinity with target protein and it could be used as potential drug as antimicrobial. Amongst all the docked compounds, compound** 8g** shows good binding affinity and interaction with topoisomerase II DNA gyrase enzymes (2XCT) with reference to norfloxacin shown in Figures 1 and 2.

## 4. Conclusion

In summary, a new series of novel 1-ethyl-6-fluoro-4-oxo-7-{4-[2-(4-substituted phenyl)-2-(substituted)-ethyl]-1-piperazinyl}-1,4-dihydroquinoline-3-carboxylic acid derivatives were synthesized and characterized by FT-IR, ^1^HNMR, and high resolution mass (HRMS-EI) spectral analyses. All the molecules were studied for their interactions with topoisomerase II DNA gyrase enzymes by molecular docking protocol. Among the tested molecules, compound** 8g** exhibited a good Glide score value of –8.51 and Glide energy −54.38 with Emodel value of  −105.2.* In vitro* antibacterial activity of the tested compounds shows improved activity against all the microorganisms used. In particular, compound** 8g** exhibits marked activity against two microorganisms. The results of antibacterial activity are supported by docking analysis. The nice docking scores of** 8g**,** 8h**,** 8i**,** 8n**, and** 8q** reveal that these compounds are well accommodated in active site of enzyme and the binding pattern of compounds** 8g**,** 8h**,** 8i**,** 8n**, and** 8q** showed that they strongly interact within the active site of topoisomerase II DNA gyrase enzymes (2XCT).

## Figures and Tables

**Table 1:** Physical data of synthesis of 1-ethyl-6-fluoro-4-oxo-7-{4-[2-(4-substituted phenyl)-2-(substituted)-ethyl]-1-piperazinyl}-1,4-dihydroquinoline-3-carboxylic acid (**8a**–**8t**).

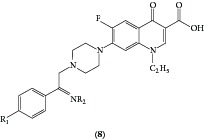								
Sr. number	Comp. number	R_1_	R_2_	Mol. wt.	Molecular formula	Yield (%)	Melting point (°C)	*R*_f_^*^

1	**8a**	–H	–NHC_6_H_5_	527	C_30_H_30_F_1_N_5_O_3_	72	189–191	0.66
2	**8b**	–H	–NH_2_	451	C_24_H_26_F_1_N_5_O_3_	50	204–206	0.39
3	**8c**	–H	–OH	452	C_24_H_25_F_1_N_4_O_4_	64	201–203	0.37
4	**8d**	–H	–NHCONH_2_	494	C_25_H_27_F_1_N_6_O_4_	54	252–254	0.41
5	**8e**	–H	–OCH_3_	466	C_25_H_27_FN_4_O_4_	60	231–234	0.54
6	**8f**	–OCH_3_	–NHC_6_H_5_	557	C_31_H_32_F_1_N_5_O_4_	70	228–230	0.60
7	**8g**	–OCH_3_	–NH_2_	481	C_25_H_28_F_1_N_5_O_4_	60	160–162	0.39
8	**8h**	–OCH_3_	–OH	482	C_25_H_27_F_1_N_4_O_5_	55	215–218	0.35
9	**8i**	–OCH_3_	–NHCONH_2_	524	C_26_H_29_F_1_N_6_O_5_	62	242–245	0.41
10	**8j**	–OCH_3_	–OCH_3_	496	C_26_H_29_FN_4_O_5_	61	235–238	0.54
11	**8k**	–CH_3_	–NHC_6_H_5_	541	C_31_H_32_F_1_N_5_O_3_	64	227–230	0.62
12	**8l**	–CH_3_	–NH_2_	465	C_25_H_28_F_1_N_5_O_3_	53	198–201	0.37
13	**8m**	–CH_3_	–OH	466	C_25_H_27_F_1_N_4_O_4_	58	242–244	0.35
14	**8n**	–CH_3_	–NHCONH_2_	508	C_26_H_29_F_1_N_6_O_4_	61	158–160	0.43
15	**8o**	–CH_3_	–OCH_3_	480	C_26_H_29_F_1_N_4_O_4_	65	202–204	0.58
16	**8p**	–NO_2_	–NHC_6_H_5_	572	C_30_H_29_FN_6_O_5_	54	228–230	0.62
17	**8q**	–NO_2_	–NH_2_	496	C_24_H_25_FN_6_O_5_	62	177–180	0.58
18	**8r**	–NO_2_	–OH	497	C_24_H_24_FN_5_O_6_	69	258–260	0.52
19	**8s**	–NO_2_	–NHCONH_2_	539	C_25_H_26_FN_7_O_6_	72	207–209	0.58
20	**8t**	–NO_2_	–OCH_3_	511	C_25_H_26_FN_5_O_6_	70	230–232	0.62

^*^Mobile phase: chloroform : hexane : methanol (9 : 0.5 : 0.5).

**Table 2:** *In vitro* antibacterial activity of synthesized compounds against Gram-positive and Gram-negative bacteria (MICs in *µ*g/mL).

Minimum inhibitory concentrations (*μ*g∖mL)							
Sr. number	Comp. number	R_1_	R_2_	*S. aureus *	*S. pneumonia *	*P. aeruginosa *	*E. coli *
				*ATCC-29213 *	*ATCC-49619 *	*ATCC-27853 *	*ATCC-25922 *
1	**8a**	–H	–NHC_6_H_5_	50	100	100	25
2	**8b**	–H	–NH_2_	25	100	100	12.5
3	**8c**	–H	–OH	50	100	100	50
4	**8d**	–H	–NHCONH_2_	12.5	100	100	25
5	**8e**	–H	–OCH_3_	50	>100	>100	50
6	**8f**	–OCH_3_	–NHC_6_H_5_	25	100	100	25
7	**8g**	–OCH_3_	–NH_2_	6.25	12.5	100	12.5
8	**8h**	–OCH_3_	–OH	6.25	50	100	12.5
9	**8i**	–OCH_3_	–NHCONH_2_	6.25	12.5	50	12.5
10	**8j**	–OCH_3_	–OCH_3_	50	100	100	50
11	**8k**	–CH_3_	–NHC_6_H_5_	50	100	>100	25
12	**8l**	–CH_3_	–NH_2_	12.5	25	100	25
13	**8m**	–CH_3_	–OH	50	100	100	50
14	**8n**	–CH_3_	–NHCONH_2_	6.25	25	50	12.5
15	**8o**	–CH_3_	–OCH_3_	25	100	>100	25
16	**8p**	–NO_2_	–NHC_6_H_5_	50	100	>100	50
17	**8q**	–NO_2_	–NH_2_	12.5	25	50	12.5
18	**8r**	–NO_2_	–OH	6.25	50	100	12.5
19	**8s**	–NO_2_	–NHCONH_2_	6.25	12.5	50	6.25
20	**8t**	–NO_2_	–OCH_3_	25	100	100	25
21	Norfloxacin			1	6.25	6.25	1

**Table 3:** Docking result of fluoroquinolones analogue.

Sr. number	Title	Docking *G*-score	Glide energy	Glide Emodel	XP H-bond	XP LipophilicEvdW	XP Electro
1	**8g**	−8.51	−54.3846	−105.214	−2.68	−4.84	−0.99
2	**8i**	−8.23	−72.1348	−119.918	−1.09	−6.14	−1.01
3	**8e**	−8.22	−70.9957	−98.0172	−1.95	−4.99	−1.27
4	**8c**	−7.95	−73.5718	−104.847	−1.83	−4.96	−1.16
5	**8s**	−7.83	−69.62	−117.139	−1.89	−5.67	−1.27
6	**8q**	−7.68	−63.5086	−106.854	−2.3	−4.98	−1.4
7	**8h**	−7.56	−74.8308	−102.867	−2.27	−4.06	−1.23
8	**8n**	−7.53	−65.1719	−95.4165	−0.68	−5.69	−1.16
9	**8a**	−7.43	−57.3206	−84.8511	−2.16	−5.19	−1.08
10	**8b**	−7.26	−69.2937	−97.1236	−2.11	−4.74	−1.41
11	**8r**	−7.26	−65.4352	−109.856	−0.81	−4.92	−1.54
12	**8p**	−6.84	−62.2759	−99.8452	−0.68	−3.76	−2.4
13	**8j**	−6.4	−69.2569	−108.236	−1.76	−4.42	−1.21
14	**8f**	−6.39	−69.32	−107.169	−0.64	−6.27	−0.49
15	**8k**	−6.31	−67.8452	−108.441	−0.04	−6.28	0.02
16	**8l**	−5.79	−68.5566	−97.1257	−0.80	−5.44	−0.35
17	**8t**	−5.57	−67.2658	−102.789	−0.64	−4.75	−1.18
18	**8m**	−5.11	−55.2368	−103.655	−0.81	−4.15	−1.15
19	**8d**	−3.86	−62.1285	−105.235	−0.8	−5.85	−1.21
20	**8o**	−3.63	−69.23	−110.249	−2.01	−4.56	−1.07

Standards were taken							

Norfloxacin		−7.29	−54.2302	−83.0725	−1.68	−4.14	−1.04
